# Potentiation Physiologically Proven

**Published:** 1893-01

**Authors:** Gustav Jaeger

**Affiliations:** Stuttgart, Germany


### POTENTIATION PHYSIOLOGICALLY PROVEN.

BY PROF. DR. GUSTAV JAEGER, STUTTGART, GERMANY.

[Translated from the Leipzig *Allgem, Homœop. Zeitung*, Vol. 124, No. 11, by B. Fincke, M. D.]

Continued from page 373.

For reasons which I shall give later, I have arranged the measurements of the Alkali salts in two parts. First, of all the salts—starting with the 3d potency—I measured one potency after another until the action turned and an animating effect appeared in the place of paralytic effects, as a sign that then the *point of indifference* was passed.

Only after having done so with 17 salts I commenced the measurement of the potencies which furnish animating effects, and this forms the second part of the measurements. I call the first period that of the *paralyzing* doses, the second that of the *animating* doses, and shall later describe every one by itself and treat also here both in particular when speaking about the quantity of error—why, will be seen hereafter.

The following Table I renders the *results of the quantities of error* in the first period, viz.:

The first column contains the date of the day of measurement, the second nominates the measured objects and especially the number of the measured potency and gives the name of the salt in abbreviation—*e. g.*, the first day the 3d potency of seven different Kali-salts was measured, the fourth day the 9th potency of two Kali-salts and the 3d potency of four Ammonia-salts, etc.

The *third* column gives the obtained rest-number in mill-seconds *seriatim.* On most days two such series are under the same date; between their execution I always paused for one hour in order to give the body time to purify itself thoroughly from the substances taken in the first series.

The *fourth* column is free for some remarks and the continuation of longer series.

The *fifth* column contains the difference between the highest and lowest rest-numbers of the series in mill-seconds.

### Table No. I. The Rest-Numbers of the First Period of Measurement.

| Date. | Measured Objects. | Rest-Numbers in Mill-Seconds. | Remarks. | Max. Diff. |
|---|---|---|---|---|
| 23/10. | 3 Potency of 7 Kali-S. | 107 107 106 108 107 107 106 | | 4 |
| 24/11. | 4 Potency " 7 " | 103 102 104 100 102 105 103 | Wood-splitting. | 5 |
| | 5 Potency " 7 " | 100 101 100 101 101 101 102 | | 2 |
| 25/11. | 6 Potency " 7 " | 104 105 102 101 104 101 101 | Wood-splitting. | 4 |
| | 7 Potency " 7 " | 102 101 101 102 102 101 102 | | 1 |
| 26/11. | 8 Potency " 7 " | 97 98 98 99 98 98 96 | | 3 |
| | 9 Potency of 2 K. 3 of 4 Am. | 102 100 98 102 100 101 | | 3 |
| 27/11. | 10 Potency " 2 " 4 " 4 " | 101 100 100 100 100 100 | | 1 |
| | 11 Potency " 2 " 5 " 4 " | 104 102 101 102 101 103 | | 3 |
| 28/11. | 12 Potency " 2 " 6 " 4 " | 100 101 100 100 100 100 | | 1 |
| 1/12. | 13 Potency " 2 " 7 " 4 " | 99 100 105 101 102 101 | | 6 |
| 2/12. | 14–16 of Kali 8 " 4 " | 102 98 99 98 96 99 100 100 100 | | 6 |
| 3/12. | 9 and 10 of 4 Ammon. | 99 101 99 99 97 100 102 100 | | 5 |
| 4/12. | 11 of 4 Ammon. | 101 101 100 100 | | 1 |
| | 12, 13, 14 of 2 Ammon. | 101 100 101 99 100 100 | | 2 |
| 5/12. | 15, 16, 17 " 2 " | 98 100 99 98 100 100 | | 2 |
| | 18, 19 2 " | 95 97 99 97 | | 4 |
| 7/13. | 3 Potency of 6 Natron-S. | 97 98 96 98 95 96 | | 3 |
| | 4 Potency " 6 " | 97 95 97 93 97 97 | | 4 |
| 11/12. | 5 Potency " 6 " | 108 107 107 106 | Not in condition. | 2 |
| | 6 Potency of 4 of 7 of 2 Nat. | 105 106 105 105 104 105 | | 2 |
| 14/12. | 8–11 Potency of 2 Nat. | 109 105 103 104 106 106 | | 6 |

I Period. In this period the *paralyzing* doses were measured. It comprises 14 days of measurement which lay between Nov. 23d and Dec. 14th. Twenty-two series were measured which together contain 137 rest-numbers. The following is remarkable :

(*a*) In the 22 series the maximal difference amounted to

| | | | |
|---|---|---|---|
| 3 | times | 6 | mill-seconds. |
| 2 | " | 5 | " " |
| 4 | " | 4 | " " |
| 4 | " | 3 | " " |
| 5 | " | 2 | " " |
| 4 | " | 1 | " " |

Since the rest-numbers are not far from 100, the differences can also be taken by per cent. and this gives for this period a quantity of error between 6% and 1%. Comparing this with the medicine-numbers of this period in the concerning tables, we perceive differences which so far overstep the highest quantity of error that the medicine-action is clearly demonstrated.

(*b*) Of the 3d potency I measured at the same day only one series, because at this concentration too strong an accumulation in the body was to be expected. The next two days (Nov. 24th and 25th) I measured two series each, but so that I occupied myself for an hour with splitting wood in open air between every first and second series in order to get rid of the eventual remainder of substances. A comparison of the series of numbers before and after this labor renders, by numbers, very nicely the daily experience that bodily labor in the open air 1. Quiets the nerves: on the first day the rest-numbers of the series before the number differed by 5 mill-seconds, on the second by 4; after the labor the difference at the first day is only 2 mill-seconds, and the second day even only 1 mill-second. 2. The quieting of the nerves is combined with an increase of velocity of work (removal of paralyzing or fatiguing substances).

On the first day the mean out of the 7 numbers of the first series is 103, that of the second 101. The rest-number, therefore, is improved by 2 mill-seconds. On the second day the difference indeed is less, viz.: only 1 mill-second, but, compared with the experience, it has a symptomatic value. Since the fourth day where the 8th potency was already extant as a substance of considerable volatility, I considered the filling out of the pause between the two series by corporeal labor no more necessary.

(*c*) On comparing the *absolute* height of the rest-numbers in the various series *three* periods are distinguished; a first of three days each (Nov. 23d, 24th, 25th) and a last of two days each with higher numbers and a middle one with shorter numbers. Thereon the following is to be said: the large numbers of the two last days of measurement are sufficiently explained by the remark on the 11th and 12th: "not in condition;" an error in diet on the 10th and 12th had influenced the disposition disadvantageously. No distinct cause can be assigned for the long numbers of the first three days, but the improving action of corporeal labor on the second and third day proves that here something is concerned which is called an "indisposition."

II Period. In this period were measured the *animating* potencies, of which the Table II gives account arranged in the same manner as Table I.

*Table II.—The rest-numbers of the second period of measurement.*

(See table, page 41.)

This table, in the interval from December 16th to February 11th, comprises 15 days of measurement with 22 series which together contain 140 rest-numbers. The maximal differences of the 22 series are:

| | | | |
|---|---|---|---|
| 1 | time | 3 | mill-seconds. |
| 5 | times | 2 | " |
| 13 | " | 1 | mill-second. |
| 3 | " | 0 | " |

Every one must admit that this expresses a surprising exactitude, if it is considered that every number is obtained by the

### Table No. II. The Rest-Numbers of the Second Period of Measurement.

| Date. | Measured Objects. | Rest-Numbers in Mill-Seconds. | Remarks. | Max. Diff. |
|---|---|---|---|---|
| 16/12. | 5 or 7 Potency of 4 Nat. | 100 100 99 100 | | 1 |
| | 7 or 9 Potency “ 5 Kali. | 100 100 99 100 100 | | 1 |
| 17/12. | 6 or 8 Potency “ 4 N and 1 K. | 100 101 100 100 100 100 101 | | 1 |
| | 10 Potency of 4 Nat and 2 K. | 100 100 100 101 100 99 | | 2 |
| 18/12. | 12 Potency “ 4 “ 2 “ | 100 100 100 100 100 99 | | 1 |
| | 14 Potency “ 4 “ 2 “ | 100 100 100 100 100 99 | | 1 |
| 21/12 | 16 Potency “ 4 “ 2 “ | 99 99 99 99 99 99 | | 0 |
| 22/12. | 18 Potency “ 4 “ 2 “ | 98 99 99 99 99 98 | | 1 |
| 23/12. | 20 Potency “ 4 “ 2 “ | 100 99 100 99 99 99 | | 1 |
| 24/12. | 22 Potency “ 4 “ 2 “ | 99 99 100 99 99 100 | | 1 |
| 28/12. | 25 Potency “ 4 “ 2 “ | 99 99 99 99 99 99 | | 0 |
| 29/12. | 30 Potency “ 4 “ 2 “ | 100 100 100 101 100 101 | | 1 |
| 30/12. | 12–22 Potency, and all 3 taken promiscously. | 99 99 98 100 98 98 99 99 | | 2 |
| | 13 Potency of 2 Am. 2 Nat. | 98 98 98 98 | | 0 |
| 4/1. | 15 Potency “ 2 “ 2 “ | 101 100 100 100 | | 1 |
| | 17 Potency “ 2 “ 2 “ 1 K. | 100 101 101 101 101 | | 1 |
| 5/1. | 19 Potency “ 2 “ 2 “ 2 “ | 100 100 100 99 100 100 | | 1 |
| | 21 Potency “ 3 “ 2 “ 2 “ | 100 101 99 100 99 100 100 | | 2 |
| 7/1. | 23 Potency “ 4 “ 2 “ 2 “ | 100 100 99 99 99 100 101 99 | | 2 |
| | 25 Potency “ 4 “ 2 “ 2 “ | 99 100 99 100 99 99 99 99 | | 1 |
| 8/1. | 30 Potency “ 4 “ 2 “ 2 “ | 98 97 97 96 98 97 97 99 | | 3 |
| 11/2. | 1000 Potency of 12 Salts. | 90 90 91 91 91 92 92 90 90 90 91 | | 2 |

addition of 4.10 acts taking off a decimal. This, therefore, means if, *e. g.*, as above the difference is only 1 mill-second for 13 times, the total sum of 40 acts gives only a difference of 10 mill-seconds. Since the clock-number is 4 mill-seconds, the difference in clock-numbers is 2.5, and since 4 decades are measured, the sums of 4 decades should not differ more than 2.5 clock-numbers. This fact at once excludes the assumption as if this could be done "arbitrarily." What may be done "arbitrarily" in measuring smallest measures of time can be shown very easily by the chronoscope if the hand, by lifting the finger, is allowed to run on with the intention of arresting it at a certain number of the dial, *e. g.*, at zero. If this is done, *e. g.*, 10 times, it, after some exercise, can happen that zero is now and then hit upon, but in the midst of it errors of 15 to 20 clock-numbers appear in both directions, too late or too early. This becomes still more irregular when the points between which the hand is to move are brought nearer together. In short, here is absolutely nothing to gain by "*corriger la fortune;*" whoever, in measuring tries to correct the numbers arbitrarily, obtains the very contrary of equalness; perfect irregularity. The former is only then to be obtained, when *every arbitrariness* is *excluded* and one understands to observe with most possible passivity and repose of spirit and will. Then, and *only then* the obtained number is the exact expression of the nerve-disposition which is independent of the will, and then one is capable of measuring by numbers even the smallest influence.

For the present investigation the result is obtained that in the animating potencies measured in the second period the limit of error is more than 1 mill-second, *i. e.*, about 1 per cent. in six out of 22 series. This is an exactness with which even an astronomer would be contented, and which in comparison with the magnitude of the values to be measured at the object oscillating between 70 per cent. minus and 90 per cent. plus, is of course abundantly large. Yea, look at any series of numbers of the later following great medicine-table, and you will find the startling fact, speaking for the potential capacity of the

Neural Analysis, that with few exceptions the difference between the numbers of two neighboring decimal potencies supersedes the quantity of error by several times.

Finally, the following fact is to be considered: In chemistry the analytical reagents fail to act entirely as soon as a substance is attenuated only as far as the 6th potency. The Neural Analysis, however, goes with certainty up to the 30th decimal potency, so that every one can be distinguished from the other and it measures yet the 1,000th potency—*i. e.*, distinguishes them with the greatest ease from nothing. In general it is quite astonishing: while with the chemist the possibility of distinguishing a *small* quantity of matter from *nothing*, *decreases* the *more*, *the smaller the quantity* and the greater the attenuation. It is the reverse with the Neural Analysis, the *higher* the *potency*, and the greater the attenuation, the easier *it can be distinguished from nothing.*

This leads us to what has been said before: the chemical action of the substance which the chemist observes in the reactions is in direct ratio to the *mass* of the molecules composing it, with it it decreases and increases. Contrariwise the living tissues react upon the *velocity* of the molecules, and since this increases with the attenuation, the reaction becomes the stronger the more the mass decreases. In the Healing Art the *volatile* substances are long since called *Nervina*, because an experience of a thousand years has taught that they act especially upon the nerves. By potentiating a substance, its volatility increases, and substances which originally are not volatile can be rendered so that they act upon the nerves just as substances originally volatile. Finally, Neural Analysis operating with the nerves is the method which makes it possible to measure the volatility of a substance according to numbers in a manner similarly as *heat* is measured by the thermometer.

On comparison of the two tables of the rest-numbers it is evident that in the second period the nerve-disposition was a much more equable one, therefore the quantity of error much less than in the first period. It may be that it was the measurement of the paralyzing doses which in the first period disquieted the nerve-disposition more because the concentrated substances did not disappear as rapidly as the animating potencies do on account of their greater volatility. It is also possible that dietetic circumstances may have influenced it. Notwithstanding, at any rate it is more agreeable that in the second period in which the so much-doubted action of the animating doses had been measured, the disposition was better, the quantity of error less, therefore the result more certain than in the first period, where the measurement brought things to light which are confirmed by further experiences.

About the *absolute height* of the rest-numbers in Table II is to be remarked that except the last day of measurement a remarkable accordance occurs—on 13 days the shortest number is 98, the highest 101, therefore a difference of only 3 mill-seconds! The 14th day (January 8th), which with the maximal difference of 3 also indicates a greater disturbance of the nerve-disposition, projects with the numbers 97 and 96, a little beyond the present frame, but the cause of it is unknown to me.

To the strikingly short numbers of the last day the following is to be remarked: Between this day and February 11th and the preceding January 8th is more time than a month, and in this time falls the conclusive measurement of the potencies of Kali-carbonicum which have been described in Part I. The main cause of the difference is that I had proceeded to the measuring *entirely sober*, therefore, without previous breakfast, and thus intentionally, in order to give to the reader a view of Neural Analysis, also from this side: Hunger is a state of excitement which characterizes itself by a shorter nerve-time, the difference against the 13 first days of measurement is almost exactly 10 per cent. It is clear that the equalness of the disposition has not been jeopardized, because in the series of 12 terms the maximal difference, therefore the quantity of error, is only 2 mill-seconds.

[TO BE CONTINUED.]

